# Encephalomyelitis associated with coronavirus disease 2019: a case report

**DOI:** 10.1186/s13256-022-03539-9

**Published:** 2022-08-23

**Authors:** Riwanti Estiasari, Kartika Maharani, Fitri Octaviana, Anyelir Nielya Mutiara Putri, Syifa Laila Ramadhan, Anna Rozaliani, Darma Imran

**Affiliations:** 1 Department of Neurology Faculty of Medicine Universitas Indonesia, Cipto Mangunkusumo Hospital Jakarta, Jl. Salemba 6, Jakarta, 10430 Indonesia; 2 Department of Parasitology Faculty of Medicine Universitas Indonesia, Cipto Mangunkusumo Hospital Jakarta, Jl. Salemba 6, Jakarta, 10430 Indonesia; 3 Cipto Mangunkusumo General Hospital Jakarta, Jakarta, Indonesia

**Keywords:** COVID-19, Longitudinally extensive transverse myelitis, Encephalomyelitis, Case report

## Abstract

**Background:**

Despite a considerable number of articles regarding neurological manifestations associated with severe acute respiratory syndrome coronavirus 2 infection, reports on transverse myelitis and encephalitis are scarce.

**Case presentation:**

We report a 35-year-old Asian Arab female presenting with longitudinally extensive transverse myelitis within 3 weeks after being diagnosed with mild coronavirus disease 2019 infection. Administration of high-dose methylprednisolone led to significant clinical improvement. However, 2 days after discharge, the patient was readmitted with encephalitis manifestations, consisting of fever and loss of consciousness, along with deterioration in myelitis symptoms. Severe acute respiratory syndrome coronavirus 2 antibody was detected in cerebrospinal fluid, but DNA of severe acute respiratory syndrome coronavirus 2 was not found. Clinical recovery was achieved after the administration of intravenous immunoglobulin.

**Conclusion:**

Longitudinally extensive transverse myelitis can be a neurological manifestation of coronavirus disease 2019 and can be followed by encephalomyelitis episodes. High-dose steroids and intravenous immunoglobulin as an immunomodulator are possible effective treatment options.

## Introduction

Neurologic involvement in patients with coronavirus disease 2019 (COVID-19) has been documented extensively, with the most prevalent self-reported neurological complaints being relatively mild, such as headache, hyposmia, and hypogeusia. Examples of uncommon nervous system disorders in this population include, but are not limited to, stroke, acute myelitis, and encephalitis [1]. Acute transverse myelitis, which by itself is a rare neurological condition, has been described to be associated with COVID-19 in various countries; however, the evidence is largely in the form of case reports or series [2, 3]. In addition, a global report showed that, among the neurological signs and/or syndromes encountered, meningitis and/or encephalitis and myelopathy were the least frequently found, occurring in 0.1% and 0.2% of hospitalized patients with COVID-19, respectively [4]. Acute disseminated encephalomyelitis (ADEM), a subtype of encephalitis correlated with poor recovery and high mortality, has also been increasingly reported in these patients [5]. In this article, we present a rare case of longitudinally extensive transverse myelitis (LETM) occurring as a parainfectious manifestation of COVID-19 followed by encephalitis in a female patient. Previous publications have noted LETM and post-COVID encephalomyelitis occurring on separate occasions, but only a few patients developed both [2, 6, 7].

## Case presentation

**Fig. 1:**
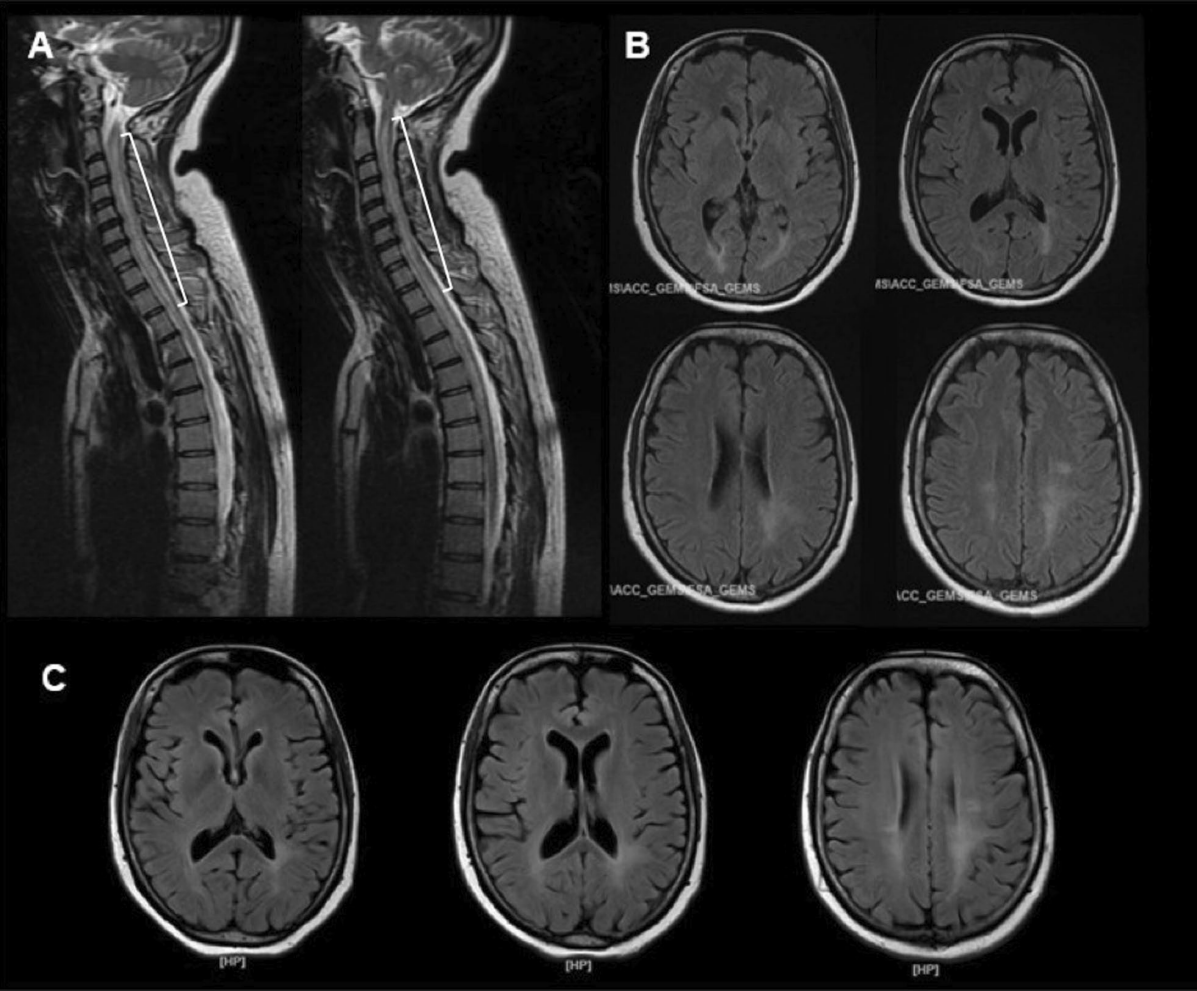
**A** Spine MRI scan before the first admission, showing T2 hyperintensity along the cervicothoracic region, corresponding with LETM; **B** brain MRI result during the first admission, revealing hyperintensity in T2 and fluid-attenuated inversion recovery (FLAIR) in the posterior horn of the lateral ventricles; **C** repeat brain MRI scan obtained during the second admission, exhibiting no new lesion

A 35-year-old female Asian Arab patient was referred to a tertiary health care facility in Jakarta, Indonesia, with transverse myelitis following severe acute respiratory syndrome coronavirus 2 (SARS-CoV-2) infection. She was previously diagnosed with mild COVID-19 (flu-like symptoms, including anosmia) and underwent self-isolation. However, 3 weeks later, she started experiencing pain in her lower extremities (hyperalgesia), followed by tingling sensation and gradual weakness in all four limbs. Within a few days, she was also unable to urinate and defecate. Her spine magnetic resonance imaging (MRI) scan showed T2 hyperintensities along the cervicothoracic region (Fig. 1A), suggestive of longitudinally extensive transverse myelitis (LETM). The patient had no significant prior medical history.

One day after her naso-oropharyngeal polymerase chain reaction (PCR) result for SARS CoV-2 was negative, the patient was transferred to our hospital. Upon neurological examination, it was found that she had tetraparesis, with more severe weaknesses in her lower extremities, hyperreflexia, and positive Babinski’s signs for both legs. She also had hypoesthesia, paresthesia, and proprioceptive disturbance below Thoracal 4 (Th4) level, as well as urinary and fecal retention. Blood evaluation revealed an elevated D-dimer level (4660 µg/L), with normal C-reactive protein and procalcitonin concentrations. Anti-aquaporin-4 immunoglobulin G (IgG) was not detected in her serum. Cerebrospinal fluid (CSF) analysis revealed lymphocytic pleocytosis (70 leukocytes/μl) and normal protein level. SARS-CoV-2, *Mycobacterium tuberculosis*, fungi, and other viruses were not detected by CSF PCR tests, while Gram and acid-fast bacillus stains also yielded negative results. Brain MRI showed paraventricular hyperintensity on T2 and FLAIR (Fig. 1B).

The patient received high-dose methylprednisolone (1 g/day) for 5 days, which was tapered off afterwards. Her motor strength improved remarkably, enabling her to stand up. However, her sensory and autonomic disturbances persisted. After 12 days of hospitalization, the patient was discharged to continue treatment in the outpatient clinic.

**Fig. 2:**
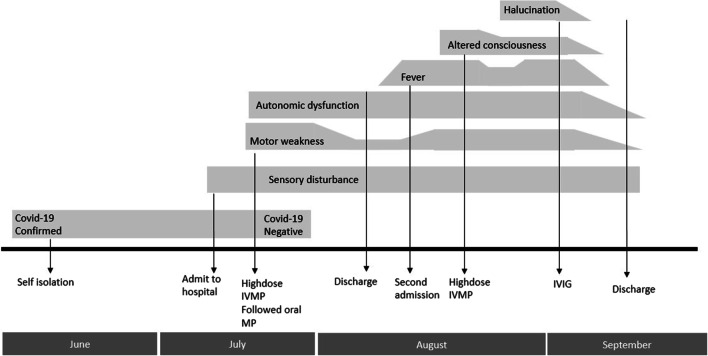
Timeline of clinical course

Two days later, the patient was readmitted to our hospital with complaints of persistent high fever (> 38 °C), nausea, and worsening of her lower limb strength (Fig. 2). Blood tests revealed normal levels of leukocytes, C-reactive protein (CRP), and procalcitonin. Meanwhile, routine urinalysis showed the presence of bacteria and nitrites, with positive urine culture for *Enterobacter* sp. (> 10^5^ colony-forming units/mL). The patient was thus diagnosed with a urinary tract infection (UTI) and treated with intravenous fosfomycin on the basis of bacterial culture and antimicrobial susceptibility testing results.

Nevertheless, her fever persisted even after 3 days of treatment with combinations of intravenous antipyretics and the use of a surface cooling device. On day 3, she tended to be in a somnolent state; we suspected the fever was not only due to the UTI, but might have a central nervous system (CNS)-underlying etiology. The patient was given 250 mg of intravenous methylprednisolone, and her consciousness improved. We performed a second brain MRI scan and found no notable changes compared with the first one (Fig. 1C). The following day, the patient was once again somnolent, accompanied by tachycardia, tachypnea, and fever, and was transferred to the intensive care unit. We escalated the methylprednisolone dose to 500 mg/day, and the patient quickly regained consciousness.

Between day 7 and day 14, the patient’s consciousness fluctuated considerably, from obtundation to a fully alert state; she even experienced disorientation and hallucinations (visual and auditory), along with intractable fever. Electroencephalography (EEG) revealed slowing background within theta frequency and intermittently high amplitude of rhythmic delta–theta wave at the bilateral frontotemporal lobe . Another lumbar puncture was performed, and CSF analysis revealed mild pleocytosis (12 cells/µL) and a positive result for anti-SARS-CoV-2 antibody (28 U/ml) with serum titer 250 U/ml. On the basis of the clinical progression and MRI and laboratory findings, the final diagnosis of COVID-19-related encephalomyelitis was made. Extensive workup did not result in any other notable findings. As high-dose corticosteroid did not provide optimal clinical resolutions, intravenous immunoglobulin (0.4 g/kg body weight) was administered for 5 days. The patient’s conditions improved substantially without any adverse event; she remained alert and did not have any hallucinations, and her EEG showed improvement, with alpha frequency dominating as background rhythm and delta waves at the bilateral frontotemporal lobe being significantly reduced (Fig. 2B). On day 23, the patient was discharged. Her autonomic functions gradually recovered, whereas no significant improvement was reported for her motor and sensory disturbances. On 4-week follow-up, she showed improvement in her motor strength and she still continued her rehabilitation program. Sometimes she remembered words that she said during her hallucination, and she was glad that it did not come up again.

## Discussion and conclusions

Inflammatory processes of the CNS due to immune response following SARS-CoV-2 infection have been recounted several times, including in encephalomyelitis. LETM is one of the neurological complications associated with SARS-CoV-2 infection, and several mechanisms underlying this condition have been suggested. First, it might be caused by direct spread of the virus to the CNS through blood circulation, nasal epithelium, or vagus nerve via angiotensin-converting enzyme 2 (ACE2) receptor, which leads to demyelination. The ACE2 receptor has been demonstrated to be the target of the COVID-19 virus and is expressed on the spinal cord membrane. Alternatively, it might be due to hyperinflammation attributable to post-infection immune response. Similar to published cases, treatment with high-dose methylprednisolone led to remarkable clinical improvements in our patient [12, 13].

The patient was readmitted to our hospital with encephalitis manifestations within 15 days after testing negative for COVID-19. Post-COVID encephalomyelitis has been described previously, although with apparently distinct presentations [7]. Positive anti-SARS-CoV-2 antibody in CSF samples of patients with COVID-19 with neurologic symptoms has also been demonstrated previously [14]. Our patient had a high titer of CSF anti-SARS-CoV-2 antibody, 28 U/mL, with a cutoff value of 0.8 U/mL. In comparison, her serum antibody titer was 250 U/mL.

Consistent with previously published reports, CSF SARS-CoV-2 tested negative by PCR, which might indicate a decreased likelihood of direct viral invasion. Nonetheless, the presence of anti-SARS-CoV-2 antibodies might be a clue to neuroinvasion (stimulated by local antigens) instead of merely reflecting circulating antibodies that enter the brain passively. A recently published laboratory study showed that CSF SARS-CoV-2 antibodies were detected only in mice with brain infections, regardless of the existence of concurrent systemic SARS-CoV-2 infection. Therefore, a positive anti-SARS-CoV-2 antibody in CSF might be highly suggestive of a SARS-CoV-2 infection of the CNS [15].

The detection of SARS-CoV-2 IgG in CSF might suggest blood–brain barrier (BBB) dysfunction and/or intrathecal IgG synthesis, but it was not possible to differentiate them in this case because we were not able to evaluate the patient’s albumin CSF and serum concentration to calculate the IgG index. We were also unable to check the interleukin (IL)-6 level, which has been suggested to play an important role in neurological disturbances related to COVID-19 and was reported to have direct association with illness severity [12].

In accordance with the proposed disease pathogenesis and pathophysiology of COVID-19, which involve cytokine and immune-mediated neuroinflammatory processes, the use of high-dose steroids, plasmapheresis, and intravenous immunoglobulin (IVIg) has been reported to be effective in patients with COVID-19 encephalopathy [16]. However, our patient showed only a modest response with high-dose methylprednisolone during the second admission. Her fluctuation in consciousness throughout the second hospitalization might be partially attributable to the administration of a relatively lower dose of methylprednisolone (250–500 mg/day). We were cautious of giving the steroid because the patient had previously received a very high dose of methylprednisolone; the risk of sepsis was also taken into consideration, as the patient had definite UTI. On the other hand, there was immediate clinical recovery following concomitant administration of IVIg therapy; a similar response in a patient with encephalopathic COVID-19 has been reported previously [16].

Taking a lesson from this case, we encourage clinicians to be aware of LETM as a manifestation of COVID-19 and be cautious of the risk of encephalomyelitis following the infection. High-dose steroids and IVIg as an immunomodulator are possible effective treatment options for encephalomyelitis associated with SARS-CoV-2 infection.

## Data Availability

Any supporting data can be obtained by direct correspondence with the corresponding author (RE).

## Abbreviations

LETM: Longitudinally extended transverse myelitis
ACE2: Angiotensin-converting enzyme 2
MP: Methylprednisolone
IVIg: Intravenous immunoglobulin
